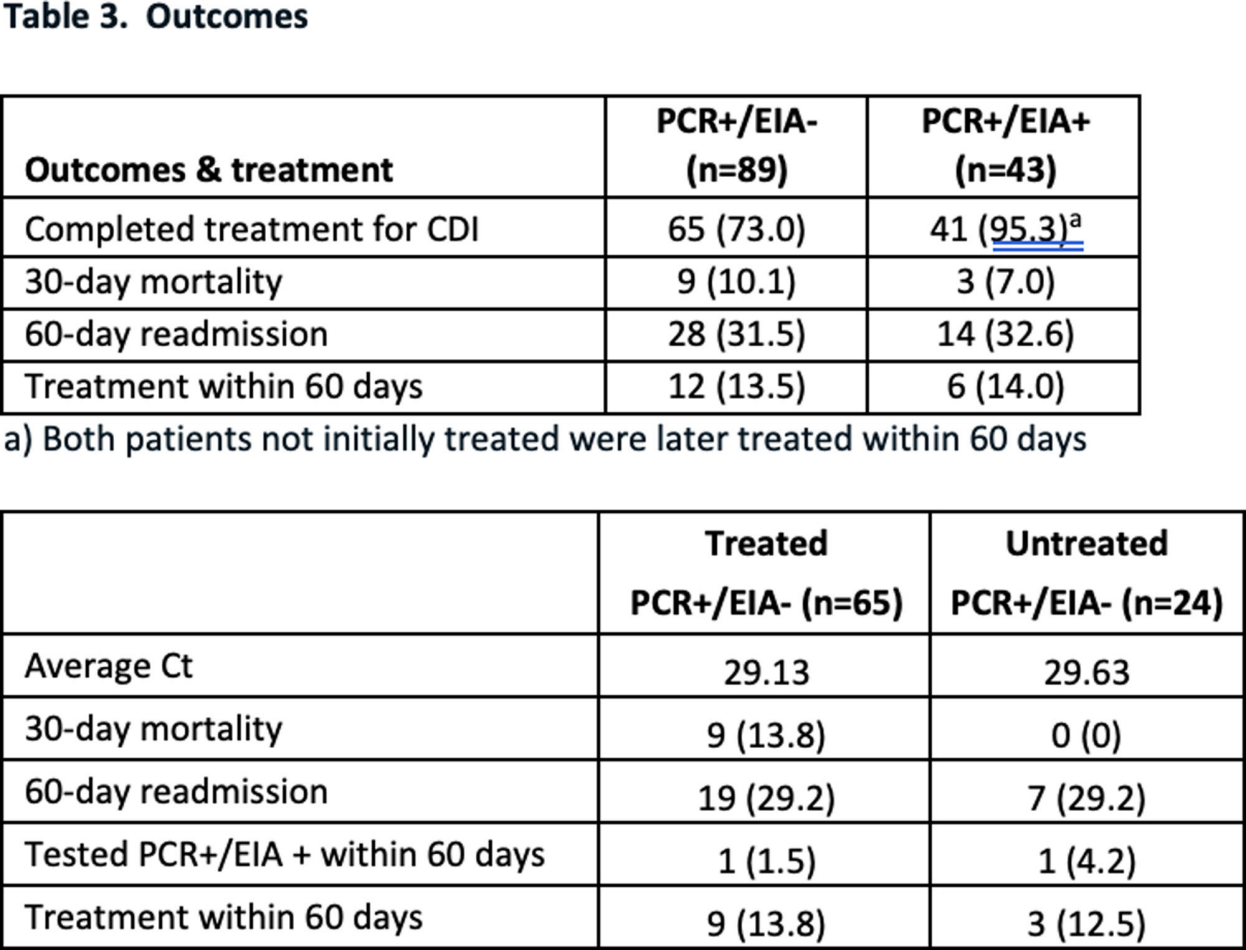# Clinical Characteristics and Cycle Thresholds Among Discordant and True Positive Test Results for Clostridiodes difficile

**DOI:** 10.1017/ash.2024.191

**Published:** 2024-09-16

**Authors:** Michael Rossi, Emerald O’Rourke, Sara Geffert, Tao Hong, Andrea Collins, Tiffany L. Chargualaf, Francine Romo Touzard

**Affiliations:** Lifespan; Warren Alpert School of Medicine; Newport Hospital

## Abstract

**Background:** The diagnosis of Clostridioides difficile infection (CDI) is challenging. Despite guideline-directed, multistep testing algorithms and diagnostic stewardship, the treatment of C. difficile colonization persists. The testing algorithm at our system utilizes an initial real-time PCR test (PCR) for Toxin B gene, which if positive, reflexes to an enzyme immunoassay (EIA) for detecting Toxins A and B. Discordant results (PCR +/EIA -) are suggestive of colonization, but the majority of patients with discordant results are treated for CDI. Correlation of C. difficile EIA B polymerase chain reaction (PCR) cycle thresholds (Ct) with the presence of free EIA and disease severity has been observed, but the ability to use Ct in the decision to treat patients with discordant results is unclear. Our study assesses if Ct values and other clinical characteristics favor treatment in select patients with discordant **Methods:** A retrospective chart review was performed of adult patients (≥ 18-year-old) with positive C. difficile PCR results that were admitted to our health system between June 01 and August 31, 2023. C. difficile PCR and Ct results were obtained by Cepheid GeneXpert and Toxin A and B EIA results were obtained by Meridian Bioscience Immunocard. Patients with discordant (PCR+/EIA) and true positive (PCR+/EIA+) results were compared. We assessed demographics, past medical history, clinical characteristics, severity of illness, PCR Ct values, treatment, and clinical outcomes including: 30-day all-cause mortality and re-admission, and 60-day CDI repeat testing and treatment. Results Of the 122 patients identified, 89 patients had discordant results and 43 had true positive **Results:** Severity of illness and other clinical and laboratory characteristics were similar between both groups. Mean Ct values were significantly lower for true positive results compared to discordant results, 24.28 vs 29.27, respectively (p26 (p=.08). Of the patients with discordant results, 73 completed treatment for CDI and no difference in clinical outcomes was observed compared to patients with discordant results that were not treated. Conclusion Ct values were lower among patients with true positive results compared to patients with discordant **Conclusion:** There were no statistically significant different rates of severe or fulminant CDI among patients with discordant results and Ct values < 26, although this finding may be limited by sample size and Ct may be helpful in deciding which discordant patients to treat.

**Figure:**
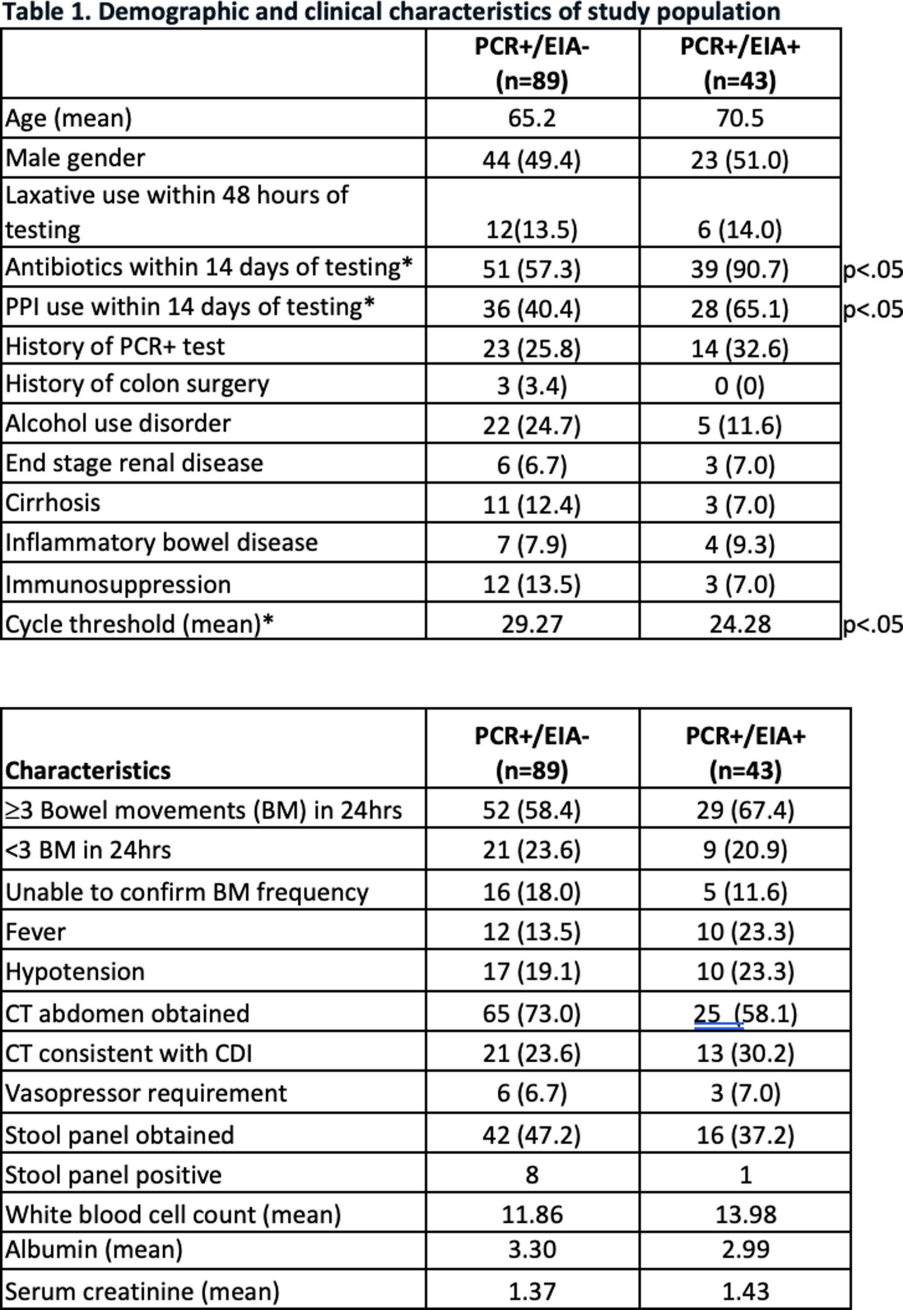

**Figure:**
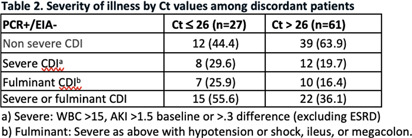

**Figure:**